# Physiological responses of coccolithophores to abrupt exposure of naturally low pH deep seawater

**DOI:** 10.1371/journal.pone.0181713

**Published:** 2017-07-27

**Authors:** Maria Debora Iglesias-Rodriguez, Bethan M. Jones, Sonia Blanco-Ameijeiras, Mervyn Greaves, Maria Huete-Ortega, Mario Lebrato

**Affiliations:** 1 Department of Ecology, Evolution and Marine Biology, University of California Santa Barbara, Santa Barbara, CA, United States of America; 2 Ocean and Earth Science, National Oceanography Centre, University of Southampton, Southampton, United Kingdom; 3 Department of Botany and Plant Pathology, Oregon State University, Corvallis, OR, United States of America; 4 Department F.-A. Forel for Environmental and Aquatic Sciences, University of Geneva, 66 Boulevard Carl-Vogt, CH, Geneva, Switzerland; 5 Department of Earth Sciences, University of Cambridge, Downing St, Cambridge, United Kingdom; 6 Department of Chemical and Biological Engineering, University of Sheffield, Mappin Street, Sheffield United Kingdom; 7 Departamento de Ecología y Biología Animal, Universidad de Vigo, Vigo, Spain; 8 Department of Geosciences, Christian-Albrechts-University Kiel (CAU), Christian-Albrechts-Platz 4, Kiel, Germany; 9 Department of Marine Ecology, GEOMAR, Düsternbrooker Weg 20, Kiel, Germany; National Taiwan Ocean University, TAIWAN

## Abstract

Upwelling is the process by which deep, cold, relatively high-CO_2_, nutrient-rich seawater rises to the sunlit surface of the ocean. This seasonal process has fueled geoengineering initiatives to fertilize the surface ocean with deep seawater to enhance productivity and thus promote the drawdown of CO_2_. Coccolithophores, which inhabit many upwelling regions naturally ‘fertilized’ by deep seawater, have been investigated in the laboratory in the context of ocean acidification to determine the extent to which nutrients and CO_2_ impact their physiology, but few data exist in the field except from mesocosms. Here, we used the Porcupine Abyssal Plain (north Atlantic Ocean) Observatory to retrieve seawater from depths with elevated CO_2_ and nutrients, mimicking geoengineering approaches. We tested the effects of abrupt natural deep seawater fertilization on the physiology and biogeochemistry of two strains of *Emiliania huxleyi* of known physiology. None of the strains tested underwent cell divisions when incubated in waters obtained from <1,000 m (pH = 7.99–8.08; CO_2_ = 373–485 p.p.m; 1.5–12 μM nitrate). However, growth was promoted in both strains when cells were incubated in seawater from ~1,000 m (pH = 7.9; CO_2_ ~560 p.p.m.; 14–17 μM nitrate) and ~4,800 m (pH = 7.9; CO_2_ ~600 p.p.m.; 21 μM nitrate). *Emiliania huxleyi* strain CCMP 88E showed no differences in growth rate or in cellular content or production rates of particulate organic (POC) and inorganic (PIC) carbon and cellular particulate organic nitrogen (PON) between treatments using water from 1,000 m and 4,800 m. However, despite the N:P ratio of seawater being comparable in water from ~1,000 and ~4,800 m, the PON production rates were three times lower in one incubation using water from ~1,000 m compared to values observed in water from ~4,800 m. Thus, the POC:PON ratios were threefold higher in cells that were incubated in ~1,000 m seawater. The heavily calcified strain NZEH exhibited lower growth rates and PIC production rates when incubated in water from ~4,800 m compared to ~1,000 m, while cellular PIC, POC and PON were higher in water from 4,800 m. Calcite Sr/Ca ratios increased with depth despite constant seawater Sr/Ca, indicating that upwelling changes coccolith geochemistry. Our study provides the first experimental and field trial of a geoengineering approach to test how deep seawater impacts coccolithophore physiological and biogeochemical properties. Given that coccolithophore growth was only stimulated using waters obtained from >1,000 m, artificial upwelling using shallower waters may not be a suitable approach for promoting carbon sequestration for some locations and assemblages, and should therefore be investigated on a site-by-site basis.

## Introduction

Upwelling is a physical process involving the wind-driven replacement of surface waters with denser, colder, nutrient-rich seawater from depth. Deep seawater contains higher nutrient, dissolved inorganic carbon (DIC) and CO_2_ concentrations than the surface ocean [1, 2]. Macronutrient concentrations can increase severalfold during upwelling [3], which enhances productivity [4–6]. The increase in DIC and CO_2_ is driven by high respiration rates in deep water masses and by increased CO_2_ solubility with decreasing temperature [1]. In some upwelling regions, such as the northeastern Pacific Ocean, changes in carbonate chemistry result in extremely high pCO_2_ values, exceeding 1,000 μatm near surface waters [2], making near-surface waters corrosive to carbonate minerals [2, 7].

In recent years, a number of geoengineering proposals have emerged in an attempt to maintain or decrease the current levels of atmospheric CO_2_. Among these, artificial upwelling has been put forward as a strategy to stimulate phytoplankton growth and, as a result, draw down atmospheric CO_2_. The main motivation is that marine phytoplankton productivity is often limited by the availability of macronutrients (nitrogen and phosphorous) or micronutrients (Fe) [8–11]. Therefore, upwelled seawater can provide these limiting nutrients and DIC into the euphotic zone, thus promoting primary production [4, 5, 12]. As a result, a number of upwelling strategies have been proposed including airlift pumps [13], and wind-/wave-powered systems [14] to increase total primary production and biomass. A consequence of artificial upwelling is that some species may be favored, for example, a shift to larger phytoplankton cells, with the potential alteration of community structure (see [15, 16]). Modeling studies have also explored the impact of artificial upwelling in carbon sequestration and the cycling of other climate relevant gases such as nitrous oxide and dimethyl sulphide, that are likely to be altered by the increase in ocean mixing [17–19].

Fertilization of surface waters benefits many taxa including diatoms and dinoflagellates [20, 21], through nutrient enrichment, although Fe limitation can be a growth-limiting factor [22, 23]. In the Arabian Sea, strong upwelling increases the magnitude of organic carbon fluxes [24], inducing exported calcite Sr/Ca changes, an indicator of productivity and calcite export flux [25]. Studies of sediment records in the Atlantic Ocean off Portugal reveal one order of magnitude increases in phytoplankton biomass dominated by diatoms during upwelling conditions (summer), while coccolithophores, on the contrary, dominate the phytoplankton community in non-upwelling conditions (winter) [26]. However, some upwelling events in the Southern Benguela upwelling [27] harbor coccolithophore blooms with high diversity of species [27]. It remains inconclusive how coccolithophore diversity and physiology will be affected by future increases in upwelling intensity.

In this study, we conducted ship deck incubation experiments with two strains of the coccolithophore species *Emiliania huxleyi* using seawater retrieved from different depths in the North Atlantic Ocean.

Through these experiments, we used a naturally acidified seawater and nutrient-rich system to test the effect of short-term abrupt exposure to deep seawater on the physiology of *E*. *huxleyi*. These experiments provide a snapshot of how the cellular and biochemical properties of coccolithophores are likely to shift in response to deep-water artificial upwelling and fertilization, relevant for geoengineering initiatives.

## Materials and methods

### Field site and incubations

Two 72-hr incubation experiments were conducted between May 26^th^ and June 4^th^ 2010 in the Porcupine Abyssal Plain (PAP) region of the central Atlantic Ocean on board RRS James Clark Ross (field permits were coordinated by the National Oceanography Center, U.K.). These short-term incubations were intended to capture abrupt rather than chronic/long term responses and to avoid large changes in carbon chemistry due to CO_2_ degassing over time and to increasing bacteria growth and respiration. The first incubation experiment (I1) was conducted using water from CTD cast 1, on May 27th 2010, at 45.47°N and 13.06°W; and the second incubation experiment (I2) used seawater recovered from CTD cast 2, conducted on May 31^st^ 2010 at 48.99°N and 16.39°W. Incubation seawater was retrieved from depths ranging from 10 to 4,831 m (Table 1) using Niskin bottles attached to a CTD rosette. Deck incubation experiments were conducted in 2.5 L polycarbonate bottles placed inside two 0.80 m^3^ transparent plexiglass tanks and maintained at 15.7 ±1°C using fast flowing natural seawater obtained from 5 m depth, to achieve 15.6–15.8°C during incubations. The incubation tanks were covered with a photoselective blue filter that maintains photosynthetic active radiation to ~10% of the surface values. This was to prevent photoinhibition (see S1 Table), and to mimic the spectral irradiance encountered by cells in the euphotic zone.

**Table 1:** Physico-chemical conditions of the seawater. Seawater was collected at the chlorophyll maximum (10 and 38 m respectively for I1 and I2); at 502 m (I1) and 508 m (I2); at 1,002 m (I1) and 1,010 m (I2); and at 4,757 m (I1) and 4,831 m (I2) for shipboard incubations with *E*. *huxleyi* strains CCMP 88E (CCMP378) (I1) and NZEH (PLY M219, CAWPO6) (I2). SW = seawater. t_0_ = beginning of the experiment; t_n_ = t_72_, 72 hours after the start of the experiment.

**I1**	***E*. *huxleyi* CCMP 88E (CCMP 378)**							
	**10 m**		**502 m**		**1,002 m**		**4,757 m**	
Temperature (°C)	15.81		11.58		10.25		2.52	
Salinity	35.81		35.63		35.85		34.89	
Oxygen (mM)	5.56		6.08		6.25		7.54	
**Incubations**	t_*0*_	t_*n*_	t_*0*_	t_*n*_	t_*0*_	t_*n*_	t_*0*_	t_*n*_
Nitrate (μM)	1.40	0.85	10.10	8.20	17.55	16.80	20.95	20.15
Phosphate (μM)	0.09	0.08	0.55	0.31	0.97	0.94	1.37	1.26
Mg/Ca_SW_ (mol/mol)	5.34 ± 0.01	-	5.31 ± 0.00	-	5.32 ± 0.01	-	5.28 ± 0.03	-
Sr/Ca_SW_ (mmol/mol)	8.57 ± 0.03	-	8.59 ± 0.01	-	8.56 ± 0.04	-	8.59 ± 0.01	-
Sr/Ca_Cal_ (mmol/mol)	-		-		-			2.85 ± 0.00
**I2**	***E*. *huxleyi* NZEH (PLY M219, CAWPO6)**							
	**38 m**		**508 m**		**1,010 m**		**4,831 m**	
Temperature (°C)	12.80		11.42		8.89		2.57	
Salinity	35.66		35.58		35.46		34.90	
Oxygen (mM)	5.97		5.56		6.45		7.53	
**Incubation 2**	t_0_	t_n_	t_0_	t_n_	t_0_	t_n_	t_0_	t_n_
Nitrate (μM)	4.90	4.80	12.25	8.55	14.25	12.55	20.90	20.50
Phosphate (μM)	0.27	0.22	0.60	0.46	0.94	0.64	1.55	1.20
Mg/Ca_SW_ (mol/mol)	5.34 ± 0.01	-	5.31 ± 0.00	-	5.32 ± 0.01	-	5.28 ± 0.03	-
Sr/Ca_SW_ (mmol/mol)	8.57 ± 0.03	-	8.59 ± 0.01	-	8.56 ± 0.04	-	8.59 ± 0.01	-
Sr/Ca_Cal_ (mmol/mol)		2.60 ± 0.02		2.57 ± 0.00		2.82 ± 0.01		2.84 ± 0.08

### *Emiliania huxleyi* strains

Monoclonal cultures of two non-axenic strains of *E*. *huxleyi* were maintained at 14°C and at 200 μmol quanta m^-2^ s^-1^ in a light-controlled incubator using a modified version of f/2 medium [28] containing 100 μM nitrate and 6.24 μM phosphate. The strains were maintained in the exponential growth phase with cell densities <100,000 cells mL^-1^ to prevent nutrient limitation and carbonate chemistry perturbations caused by calcification and photosynthesis. Strain CCMP 88E (CCMP 378), which originates from the Gulf of Maine (43.00°N, 68.00°W), was obtained from the National Center for Marine Algae and Microbiota (NCMA) (Bigelow, ME, U.S.A.). *Emiliania huxleyi* strain NZEH (PLY M219, CAWPO6) isolated from SW New Zealand waters (46.58°S, 168.05°E) was obtained from the Plymouth Culture Collection of Marine Algae (Plymouth, U.K.). We studied cell specific growth rate (μ, d^-1^), coccolith morphometrics, and biogeochemical parameters including cellular and production rates of particulate inorganic and organic carbon (PIC and POC), particulate organic nitrogen (PON), and PIC:POC and POC:PON ratios under the conditions of the two deepest water masses used. Additionally, strain-specific coccolith elemental composition of calcite Sr/Ca was investigated.

### Incubation experiments

Seawater was collected from Niskin bottles deployed from a CTD to supply seawater for incubations 1 (I1) and 2 (I2). Niskin bottles were triggered at 10, 502, 1,002, and 4,757 m for I1, and at 38, 508, 1,010, and 4,831 m for I2 (Table 1). Hereafter, we refer to these depths as chl max (chlorophyll maximum), 500 m (representing 502 m and 508 m of I1 and I2 respectively), 1,000 m (representing 1,002 m and 1,010 m of I1 and I2 respectively), and 4,800 m (representing 4,757 m and 4,831 m of I1 and I2 respectively). Prior to the incubations, seawater was filtered through a Spectrum nylon mesh of 300 μm to remove zooplankton and suspended particles. Seawater was gently filtered through 0.22 μm Polycap 36 AS disposable filters (Whatman, Little Chalfont, U.K.). Filtration was aimed to minimize the effect of biotic interactions and metabolic activity of other microorganisms on the seawater chemistry. Filtered seawater was subsequently placed in 2.5 L polycarbonate bottles, which were filled leaving a negligible headspace to minimize gas exchange. Once temperature equilibrated in the incubations (~ 1 h), *E*. *huxleyi* cells were inoculated to achieve densities < 5000 cells mL^-1^. The inoculation procedure was conducted quickly to minimize gas exchange and bottle caps were tightly sealed immediately after inoculation. Incubations were conducted for 72 hours and samples were collected at the start (t_0_) and end (t_72_) of the experiment. Each experimental condition was incubated in tripicate and a blank (without cells) was used as a control to monitor changes in carbonate chemistry in abiotic conditions during the course of the experiment.

### Physico-chemical analyses of seawater

Seawater samples were collected in triplicate at the start (from the Niskin bottle) and end of the incubations for temperature, salinity, and oxygen. Samples were collected for macronutrients (nitrate and phosphate) by syringe filtration of 15–20 mL through a 0.22 μm Millex filter (Millipore, Billerica, MA, USA) and stored at -20°C. Nitrate and phosphate concentrations were determined colorimetrically following [29] using a spectrophotometer (Hitachi U-2000 Scientific Instruments) at the Helmholtz Centre for Ocean Research Kiel (Germany). Samples for carbonate chemistry, specifically total alkalinity (TA) and dissolved inorganic carbon (DIC) were collected in 300 mL borosilicate flasks directly from the Niskin bottle (pre-filtered gently through a 0.22 μm polycarbonate capsule), and at the end of the incubations. Samples were preserved with 750 μL 3.5% HgCl_2_ solution to prevent microbial growth during storage in the dark at room temperature. TA and DIC were measured using a Versatile Instrument for the Determination of Titration Alkalinity (VINDTA) at the National Oceanography Centre (Southampton, UK). Certified reference materials (CRM) to calibrate and establish correction factors for VINDTA measurements were obtained from Dr. Andrew Dickson at the Marine Physics Laboratory of the Scripps Institute of Oceanography, University of California San Diego, USA. VINDTA-derived values for TA and DIC were corrected for various parameters including titration acid volume, nutrient concentration of the sample, temperature, salinity and CRM values. Carbonate chemistry parameters were calculated from in situ temperature, salinity, DIC, TA and nutrients using the "CO2SYS" macro [30]; for model constraints see [31] (Table 2, S2 Table).

**Table 2:** Inorganic carbon chemistry parameters. In the incubations using the two deepest water conditions, initial (t_0_) and final (t_72_) conditions are provided. First row indicates the values of the water at t_0_, immediately after filtration; the second row indicates the values at the end of the experiment (t_72_); the third row represents one standard deviation; and the last row represents the values in the blank (seawater treated in an identical way but without cells). TA, DIC, HCO_3_^-^, CO_3_^2-^ and CO_2_ values are in μmol kg seawater^-1^.

**CCMP 88E (I1)**	**TA**	**DIC**	**pH**	**[HCO**_**3**_^**-**^**]**	**[CO**_**3**_^**2-**^**]**	**[CO**_**2**_**]**	**Ω-cal**	**pCO**_**2**_ **(p.p.m.v)**
4,800 m (t_0_) (t_72_) Blank	2340.87 2340.79 (2.70) 2348.70	2203.39 2169.24 (17.73) 2187.80	7.90 7.92 (0.04) 7.898	2070.62 2018.48 (25.69) 2041.90	108.37 130.12 (10.07) 123.80	24.40 20.64 (2.04) 22.10	2.58 3.10 (0.24) 2.95	595.77 574.38 (56.71) 614.40
1,000 m (t_0_) (t_72_) Blank	2343.43 2369.96 (2.08) 2375.10	2181.75 2187.23 (1.71) 2188.90	7.93 7.94 (0.00) 7.943	2037.11 2029.99 (1.51) 2029.80	123.14 137.27 (0.43) 139.50	21.50 19.97 (0.06) 19.60	2.92 3.25 (0.01) 3.31	557.05 558.72 (1.63) 549.60
500 m	2346.25	2135.83	8.00	1964.95	154.20	16.69	3.66	471.87
Chl max	2347.37	2113.76	8.07	1931.36	167.92	14.49	3.98	391.27

**NZEH** **(I2)**	**TA**	**DIC**	**pH**	**[HCO**_**3**_^**-**^**]**	**[CO**_**3**_^**2-**^**]**	**[CO**_**2**_**]**	**Ω-cal**	**pCO**_**2**_ **(p.p.m.v)**
4,800 m (t_0_) (t_72_) Blank	2334.05 2323.25 (1.46) 2348.18	2193.30 2160.62 (5.21) 2191.45	7.91 7.90 (0.01) 7.88	2059.53 2014.54 (7.56) 2047.27	109.97 124.53 (2.97) 121.44	23.80 21.55 (0.53) 22.74	2.62 2.97 (0.07) 2.90	581.02 609.89 (7.52) 639.23
1,000 m (t_0_) (t_72_) Blank	2356.85 2293.18 (4.87) 2342.73	2187.46 2129.32 (5.07) 2178.01	7.95 7.91 (0.01) 7.89	2038.69 1984.11 (4.91) 2029.88	127.97 124.27 (0.13) 126.31	20.81 20.94 (0.24) 21.81	3.04 2.95 (0.00) 3.00	539.71 584.39 (16.76) 623.36
500 m	2346.42	2140.53	7.99	1972.06	151.34	17.14	3.59	484.71
Chl max	2352.50	2108.82	8.08	1920.56	174.43	13.83	4.14	373.71

### Seawater elemental composition

Aliquots of 25 mL seawater were filtered through a Millex 0.22 μm filter (Millipore, USA) and stored at -20°C for elemental analysis. Seawater Mg/Ca and Sr/Ca ratios were determined separately by the method of standards addition in culture medium samples diluted to 1/200 and 1/10 respectively, and measured with a Thermo iCap 6300 Series ICP spectrometer (Department of Geology, University of Oviedo, Spain). For trace element ratios, measurements were conducted both in radial and axial mode: Ca (315 nm radial) and Sr (407 nm radial). Calibrations were performed with multi-element standards offline using the intensity ratio method of [32].

### Growth rate, cell density and coccolith morphometrics

Cell counts for each incubation were made in triplicate using a Leo 1450VP scanning electron microscope (SEM) (Carl Zeiss, Cambridge, UK) in conjunction with SmartSEM software (Carl Zeiss) following the methods outlined by [33]. In brief, triplicate samples of 200-mL incubation water containing coccolithophores were gently filtered onto 25 mm cellulose nitrate filters and dried overnight at 37°C. Samples were subsequently stored in airtight containers containing silica gel to prevent degradation. Filters were mounted on aluminum stubs, gold-sputtered and a predefined transect macro was used to take 225 images (each equivalent to 1 mm^2^ area) at 5000x magnification. For each filter, images were chosen at random and counts were made until at least 300 coccospheres were enumerated. Typically, 400–2000 coccospheres were counted for each sample from 100–225 randomly selected images. Numbers were then back extrapolated to determine coccosphere density. Growth rate was determined with a standard exponential growth equation [34]: μ = [Ln (N_t_)—Ln (N_0_)] / t, where N_t_ and N_0_ are the cell densities at the start and at the harvest day respectively, and t corresponds to the length of incubation (in days). We performed one-way ANOVA using the program StatPlus:mac LE (2015 AnalystSoft Inc., 2015). Statistical significance was determined based on a *p*-value threshold of 0.05.

Coccosphere volume (cell and surrounding coccoliths) and the morphology of coccoliths were determined using SEM protocols described above. Distal shield length and area of the coccolith, central area surface (inner part of the coccolith enclosed by the rim) and coccolith circularity [35] were measured using at least 48 randomly selected coccoliths for each filter.

### Particulate elemental analyses

At the end of the incubation (t_72_), 200 mL of incubation sample was filtered through a pre-combusted GF/F filter (Whatman, U.K.) and stored at -20°C until used for analysis of cellular particulate organic carbon and nitrogen (POC and PON). Upon thawing, samples were fumed with H_2_SO_3_ for 48 hours in a dessicator chamber to remove inorganic carbon [36]. The filters were subsequently dried at 60°C for 48 hours and pelleted in pre-combusted aluminium foil (EMA 100 x 30 mm circles, following [37]). POC and PON were analysed by Mr Robert Head at Plymouth Marine Laboratory using a Thermo Finnigan Flash EA1112 elemental analyzer with acetanilide standards.

In order to assess cellular calcification content and production rates, 200 mL of the incubation sample were filtered through a 0.22 μm Nucleopore polycarbonate filter (Whatman, U.K.) that was previously rinsed with 5 mL of a diluted ammonium hydroxide solution (pH ~ 9.5). After filtration, samples were washed three times with this solution to remove salts and prevent any calcite dissolution and samples were then stored at -20°C. Prior to analysis, filtered calcite was dissolved in ~20 mL 0.1 M HNO_3_ and [Ca^2+^] was subsequently determined at the University of Cambridge (UK) using a Varian Vista Pro inductively coupled plasma emission spectrophotometer (ICP-OES; Agilent, Stockport, UK). Instrument calibration followed procedures used for the determination of Mg/Ca in foraminiferal calcite [32]. [Na^+^] was measured as a proxy for residual seawater Ca^2+^. Filters rinsed with the control medium were also analyzed and confirmed that seawater contribution to measured [Ca^2+^] was negligible. The content of Ca^2+^ per filter and per coccolithophore (using cell density) was then calculated and extrapolated to PIC, assuming that all Ca^2+^ on the filters was derived from calcite [38].

### Coccolith elemental composition

One L of sample from each incubation bottle was filtered through a 0.22 μm Nuclepore filter (Whatman, U.K.) to determine the elemental composition of coccoliths at the end of each incubation. Filters were stored in 50 mL Falcon tubes at -20°C until further treatment. After thawing at room temperature (24 h), the filters were carefully removed with Teflon tweezers, leaving behind only seawater and calcite. This material was concentrated into cellular pellets by centrifugation at 1970 x g for 20 minutes at 4°C. After discarding the supernatant, the calcite pellets were frozen at -80°C, freeze dried for 48 hours [39], and kept at room temperature until further treatment.

Pellets were cleaned following the optimized protocol in [40] and elemental analysis was performed using a Thermo iCAP 6300 Series ICP Spectrometer (Thermo Fisher Scientific, Waltham, MA, USA). Samples were diluted to a common Ca level, seeking the highest possible Ca concentration within the range of standard calibration solutions (15, 50, 100 p.p.m. Ca). For trace element ratios, we measured in both radial and axial mode: P (177 nm axial), Fe (259 nm radial), Ca (315 nm radial) and Sr (407 nm radial). Calibrations were performed with multi-element standards offline using the intensity ratio method of [32].

## Results

### Physiological responses of *Emiliania huxleyi* strains to deep-water incubations

Incubations with strains CCMP 88E and NZEH using water from the chl max and from 500 m failed to promote growth over 72 hours, i.e., cell density remained unchanged (<3% decrease in I1) or decreased (21% at the chl max and 89% at 500 m), and cell divisions were only observed in incubations with water from 1,000 m and 4,800 m. The trends in growth rate, and cellular quotas and production rates for PIC and POC, and the cellular PON values in CCMP 88E were comparable between incubations using water from 1,000 and 4,800 m (Table 3). However, PON production rates were threefold higher in the incubation with water from 4,800 m, and the POC:PON ratio was three times lower at 4,800 m compared to 1,000 m (Table 3). In strain NZEH, a significant effect of seawater depth on cellular physiology was observed. Cells of this strain displayed a decline in both growth and PIC production rates and a significant increase in cellular standing stocks of PIC, POC and PON when incubated in water obtained from 4,800 m compared to water from 1,000 m. The production rates of both POC (~0.5 pmol cell^-1^ d^-1^) and PON (0.04 pmol cell^-1^ d^-1^), as well as cellular ratios of PIC:POC (0.9) and POC:PON (11) were comparable at the end of these short-term incubations in water from 1,000 and 4,800 m (Table 3).

**Table 3:** Physiology of the *E*. *huxleyi* strains (CCMP 88E and NZEH) incubated in seawater from 1,000 m and 4,800 m for 72 hours. μ (d^-1^) represents growth rate, PIC, POC and PON are particulate inorganic carbon, particulate organic carbon and particulate organic nitrogen respectively, and values are reported in pmols. Pairwise differences were considered significant at a threshold of p<0.05, following one-way ANOVA. The trend represents neutral response (−), increase (↑), and decrease (↓) in the parameters measured in incubations using water from 4,800 m compared to those using water from 1,000 m.

Strain	SW depth	μ (d^-1^)	PIC cell^-1^	PIC cell^-1^ d^-1^	POC cell^-1^	POC cell^-1^ d^-1^	PON cell^-1^	PON cell^-1^ d^-1^	PIC:POC ratio	POC:PON ratio
**CCMP** **88E** **(I1)**	1,000	0.24 (0.06)	0.258 (0.03)	0.063 (0.02)	1.132 (0.67)	0.273 (0.19)	0.039 (0.01)	0.009 (0.00)	0.279 (0.14)	21.80 (3.61)
	4,800	0.441 (0.23)	0.311 (0.02)	0.141 (0.08)	0.491 (0.19)	0.201 (0.12)	0.070 (0.03)	0.026 (0.01)	0.729 (0.38)	7.421 (1.51)
	Trend	−	−	−	−	−	−	↑	−	↓
**p-value**		0.22	0.08	0.17	0.18	0.60	0.20	0.08	0.12	0.02*
**NZEH** **(I2)**	1,000	0.570 (0.03)	0.837 (0.03)	0.477 (0.01)	0.879 (0.03)	0.501 (0.02)	0.082 (0.00)	0.047 (0.00)	0.952 (0.01)	10.77 (0.41)
	4,800	0.304 (0.01)	1.356 (0.03)	0.412 (0.02)	1.536 (0.21)	0.467 (0.07)	0.144 (0.02)	0.044 (0.01)	0.894 (0.12)	10.71 (0.69)
	Trend	↓	↑	↓	↑	−	↑	−	−	−
**p-value**		0*	0*	0.02*	0*	0.50	0*	0.60	0.46	0.90

**Fig 1:**
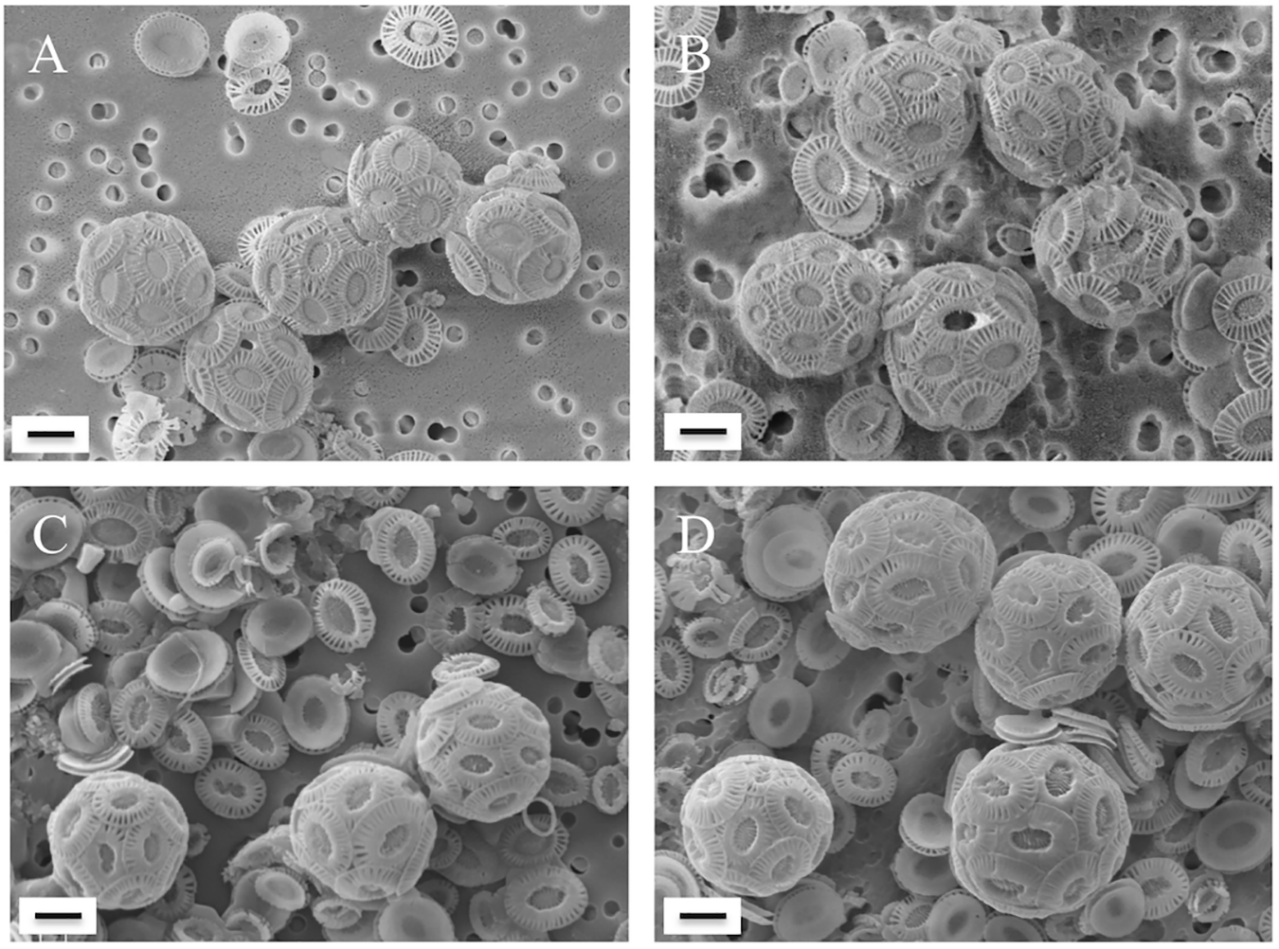
**Scanning electron microscopy images of *E*. *huxleyi* CCMP 88E (A, B) and NZEH (C, D) after incubations in water collected from 1,000 m (A, C) and 4,800 m (B, D).** Scale bar: 2 μm.

Morphometric analysis revealed that in strain NZEH, the coccosphere volume was 30% larger when incubated in water from 4,800 m compared to 1,000 m (Table 4, Fig 1). There were, however, no differences in coccolith distal shield length and surface, central area surface and coccolith circularity for cells incubated at 1,000 m and 4,800 m (Table 4, Fig 1). Strain 88E exhibited no significant differences for any of the morphometric parameters tested (Table 4, Fig 1).

**Table 4:** Morphometric analysis of *E*. *huxleyi* CCMP 88E and NZEH after incubations in water collected from 1,000 m and 4,800 m. Coccosphere = cells and surrounding coccoliths; DS = distal shield; CA = central area; DSL = distal shield length. The numbers on the second and third rows represent the standard deviation and population size respectively.

Strain	Water depth (m)	Coccosphere volume (μm^3^)	DS coccolith surface (μm^2^)	CA coccolith surface (μm^2^)	Coccolith circularity	Coccolith DSL (μm)
CCMP 88E	1,000	75.52 22.579 48	5.295 1.33 172	0.643 0.242 172	0.911 0.033 172	2.784 0.453 172
	4,800	82.756 20.257 61	5.267 1.148 233	0.651 0.225 233	0.908 0.036 233	2.835 0.325 233
	p-value	0.08	0.82	0.89	0.49	0.35

NZEH	1,000	113.506 32.767 76	5.911 1.637 177	0.849 0.347 177	0.884 0.023 177	3.07 0.378 177
	4,800	162.275 39.187 72	6.174 1.718 148	0.887 0.272 148	0.889 0.028 148	3.121 0.39 148
	p-value	<0.001*	0.15	0.27	0.12	0.23

### Seawater inorganic carbon chemistry

Seawater from different depths yielded a distinct vertical gradient of inorganic carbon species. Water from 4,800 m had comparable TA values to waters obtained from the chl max (<1% difference), but this deeper water contained up to 4% more DIC. Additionally, it had a ~7% higher concentration of bicarbonate ions ([HCO_3_^-^]), ~40% lower concentration of carbonate ions ([CO_3_^2-^]), ~40% higher [CO_2_] and ~35% higher pCO_2_. Values on the total scale pH were 0.17 units lower and omega calcite (Ω_cal_) was ~1.5 units lower at 4,800 m compared to water from the chl max (Table 2, S2 Table).

Both biological activity and calcite particles can interfere with carbonate chemistry analyses, so we gently filtered the water used in the incubations through 0.22 μm prior to commencing incubations. Our results indicate TA varied by <2% and DIC varied by <1% before and after filtration (S3 Table). [CO_2_] and [CO_3_^-2^] varied by less than 1% except in the deepest water in I1 (~5%) and I2 (~10%) and in water from the chl max in I1 (S2 Table). The pH was identical except in the deepest seawater treatment in I1, where pH decreased by 0.05 units after filtration. For I2, pH only decreased as a result of filtration (0.04 units) for water obtained from 4,800 m and for water originating from the chl max (0.02 units). After filtration, Ω_cal_ decreased by less than 0.05 units except for water obtained from 4,800 m (I1 water was 0.15 units lower after filtration; I2 was 0.3 units lower), and for water from the chl max, which decreased by 0.16 units after filtration during I1. Overall, perturbations in carbonate chemistry caused by filtration were therefore minimal (S3 Table). TA and DIC remained largely unaltered at the end of the incubation for the controls, but there were modest changes in pH, [HCO_3_^-^], [CO_3_^2-^], [CO_2_] (Table 2). Ω_cal_ varied by 0.02–0.57 units (Table 2).

The TA and DIC in incubations varied by <3% between t_0_ and t_72_ (Table 2). By the end of I1 (experiments with CCMP 88E), pH values had increased by up to 0.02 but for I2 (experiments with NZEH), pH values decreased by 0.01 (4,800 m) and 0.04 (1,000 m). [HCO_3_^-^] decreased by <3% for both incubations and for seawater obtained from all depths. During I1, [CO_3_^2-^] increased by up to 17% but in I2, only the deepest water showed an increase (12%) in [CO_3_^2-^] whereas water from 1,000 m showed a decrease in [CO_3_^2-^] of 3%. In I1, [CO_2_] decreased by up to 15% (4,800). In I2, [CO_2_] decreased (9%) in incubations with water from 4,800 m only, but remained unaltered in incubations with water from 1,000 m. The Ω-cal values in I1 increased by 0.52 and 0.33 in water from 4,800 m and 1,000 m respectively. However, in I2, Ω-cal increased only in water from 4,800 m (by 0.35 units), whereas Ω_cal_ decreased by 0.09 in the 1,000 m treatments (Table 2).

### Nutrients, seawater elemental analysis and physico-chemical conditions

Nutrient concentrations increased with depth and, with the exception of water obtained from the chl max at t_0_ for I1, macronutrients (nitrate and phosphate) were replete at both incubation time points (Table 1). Although waters from the chl max and from 500 m did not promote growth, nitrate removal was 20–40% in I1 and 2–30% in I2, and phosphate removal was 11–43% in I1 and 18–23% in I2 (Table 1). By t_72_, nutrient utilization by CCMP 88E caused a nitrate decrease of 4% and a 3% (1,000 m) and 8% (4,800 m) decrease in phosphate between the start and the end of incubations. During I2 (strain NZEH), there was a 12% (1,000 m) and 2% (4,800 m) decrease in nitrate and a 32% (1,000 m) and 22% (4,800 m) decrease in phosphate between the beginning and the end of the incubation (Table 1). Seawater elemental ratios remained constant with depth. Salinity, temperature and oxygen in the water column followed typical open ocean trends; data are given in Table 1.

### Coccolith geochemistry

**Fig 2:**
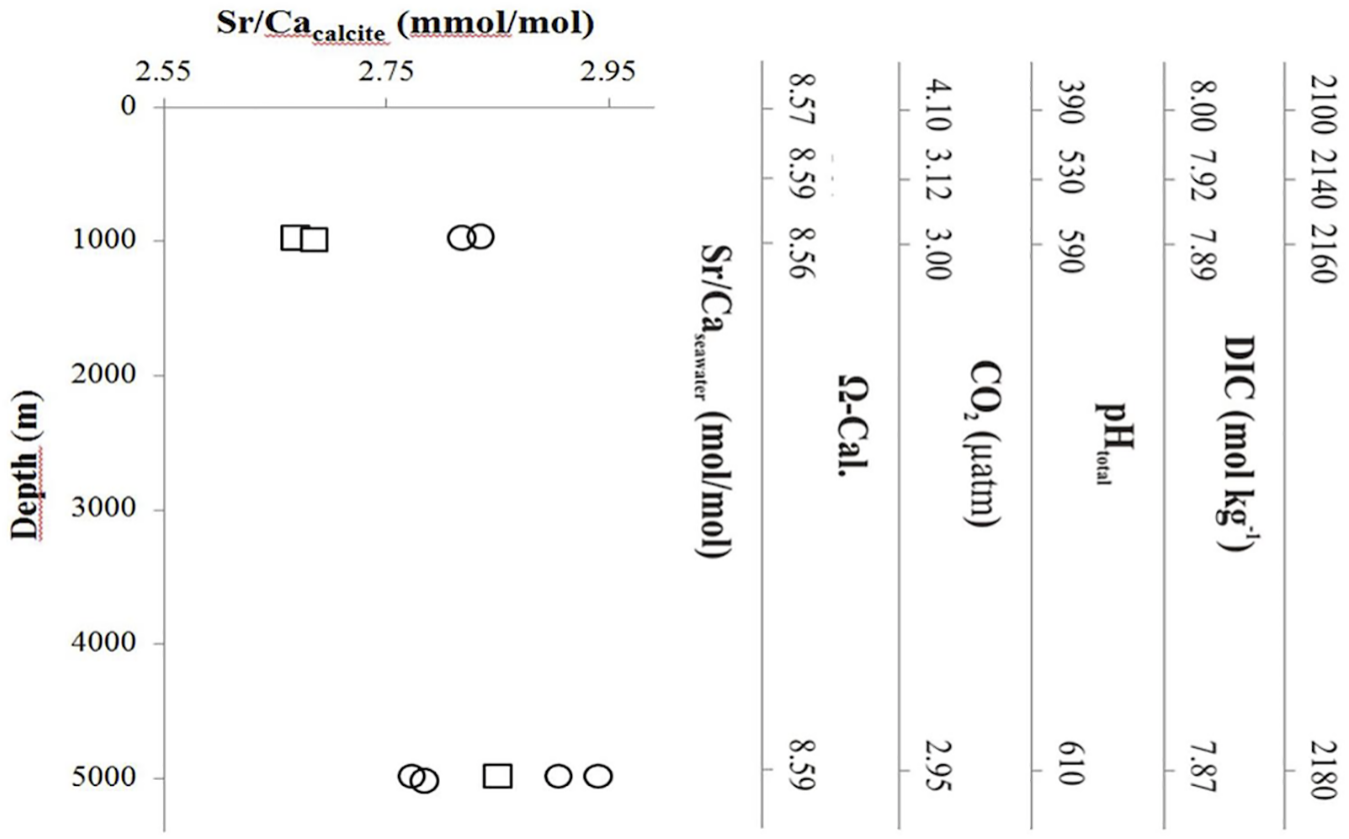
The relationship of *E*. *huxleyi* strains (CCMP 88E: circles, and NZEH: squares) calcite Sr/Ca with the seawater conditions from different depths and thus a natural carbonate chemistry gradient. Axes on the right show experimental medium carbonate chemistry conditions and seawater composition in the incubations (no pressure effect). Each data point represents an individual measurement.

Coccolith calcite Sr/Ca ratios rapidly increased from surface waters to 1,000 m and 4,800 waters, for both strains (Fig 2). The changes in the absolute calcite Sr/Ca ratio value were strain-dependent, with a larger increase in strain NZEH compared to CCMP 88E.

## Discussion

### Variable physiological responses to upwelled waters within *E*. *huxleyi*

Our results revealed lack of growth in two strains of *E*. *huxleyi* using water from the two shallowest depths, suggesting that geoengineering approaches promoting shallow water upwelling may not always be suitable to enhance productivity. Additionally, we observed differential physiological responses within the *E*. *huxleyi* species complex to abrupt changes in seawater chemistry simulating artificial upwelling. Although most artificial upwelling is from a few hundred meters (e.g., [15, 41, 42], simulations of artificial upwelling have been presented for 1,000 m [18, 19] and deeper (Atmocean, Inc, https://atmocean.com/). The depths targeted in this study correspond to those proposed by [18] and [19] and we included an additional depth of 4,800 m in an effort to expand the depth range to its maximum at this site. The deepest waters used in our study had nitrate and phosphate values comparable to those used in artificial upwelling tested in the field [15] and the levels of CO_2_ were within the range of those observed in upwelled waters enhancing primary productivity [43, 44]. There are however potential logistical drawbacks of using deep waters and more research needs to be conducted on the feasibility of deep artificial upwelling.

Despite N:P values not deviating far from the Redfield ratio (~13:1–20:1), and macronutrient excess (except for water obtained from the chl max of I1) (Table 1), growth was only promoted in water from >1,000 m (Table 3). However, although *E*. *huxleyi* did not grow in incubations using water from the two shallowest depths (chl maximum and 500 m), nutrient removal was significant (up to 40% and 30% nitrate removal in I1 and I2 respectively, and up to 43 and 23% phosphate removal in I1 and I2 respectively), indicating possible microbial competition for nutrients in our non-axenic cultures. Deep seawater is rich in nutrients and DIC and the synergistic effect of nutrients can be greater than the effect of DIC change alone in *E*. *huxleyi* [45]. In our study, DIC was non-limiting but growth was promoted only at concentrations of nitrate greater than 14 μM (Tables 1 and 3).

With the exception of the very low PON production rate in the incubation with water from 1,000 m, which gave a very high POC:PON ratio (21.8), the carbon physiology of the Gulf of Maine strain (CCMP 88E) appeared to be unaffected by the chemistry of the waters that promoted growth (1,000 m and 4,800 m). This deviation from Redfield (POC:PON ~6.6) has been reported for nutrient-replete exponentially-growing coccolithophores including *E*. *huxleyi* (POC:PON ratio = 26) and *Gephyrocapsa oceanica* (POC:PON ratio = 32) [46]. Experiments using natural populations have indicated that under conditions of nitrate repletion, POC:PON ratios can be as high as 17 under Fe scarcity and up to 37 when Fe was added to the incubations [47]. Although we are unable to speculate about micronutrient availability in the current experiment, all these studies appear to suggest a great deal of plasticity in POC:PON ratios originating from taxonomic diversity but also environmental conditions although the specific factor governing these ratios remain unclear. In a global survey, [48] reported they were unable to explain 80% of their data based on traditional oceanographic parameters and argue that other factors, or yet unexplored, may influence the C:N ratio in different ocean environments [48].

Although the decrease in DIC:DIN ratio in water from 1,000 m to 4,800 m was moderately larger in I2 (32%) than in I1 (15%), why nitrogen rather than carbon acquisition was favored to such extent in the waters with a higher content of DIC (4,800 m) in I1 remains unclear. On the other hand, the carbon physiology of strain NZEH was significantly different when cells were incubated in water from 1,000 m and 4,800 m (Table 3). The intraspecific response that we report in *E*. *huxleyi* is likely a result of inherently distinct physiological properties (e.g., growth rates and calcification) of individual strains/genotypes/morphotypes that have already been observed in lab experiments [49, 50]. This is supported by the recent sequencing of multiple *E*. *huxleyi* strains that revealed genomic differences in gene content and in genome size [51]. Field observations support this large heterogeneity of biological properties within *E*. *huxleyi*. Specifically, high latitudes are characterized by largely monospecific bloom populations [52], which display huge intraspecific genetic diversity and high productivity [52, 53]. In contrast, high species diversity and low-productivity of coccolithophore populations are characteristic of oligotrophic stratified waters of low and mid-latitudes [54].

Increasing CO_2_ levels generally enhance photosynthetic carbon fixation in *E*. *huxleyi* but, in some strains, a rise in CO_2_ appears to impact growth ([31, 55]; this study with NZEH). Also, elevated CO_2_ is known to have variable effects on coccolithophore calcification depending of strain and life cycle stage [31, 49, 55, 56, 57, 58, 59]. Although there are scarce reports from the field measuring selection under different carbon chemistry scenarios, recent field observations provide strong indication of strain selection within *E*. *huxleyi*, showing that an overcalcified morphotype of *E*. *huxleyi* (morphotype ‘R’) may be adapted to high CO_2_ upwelled waters of the Patagonian Shelf [60]. One of the strains tested in this study, NZEH, displays morphotype R characteristics and grew most rapidly in the incubations with deep seawater. This strain has been frequently used in laboratory studies [31, 45, 55, 57, 61], and in agreement with our previous results, cellular calcification (Table 3) and coccosphere volume (Fig 1, Table 4) increased under elevated CO_2_ [31, 55].

The documented intraspecific diversity in *E*. *huxleyi* physiology [49] and the environmental selection of strains [60, 62] determine the extent to which populations respond to changes to environmental pressure, including alterations in CO_2_. However, population responses may differ depending on whether the environmental perturbation is abrupt or chronic. Upwelling represents an analogue of exposure to both abrupt short-term and long-term stress that could have consequences that go far beyond a cellular response but extend to other levels of biological organization [63, 64]. At the cellular level, acclimation to abrupt environmental change is usually initiated by a stress response characterized by transitory molecular and physiological perturbations to maximize resource utilization while maintaining structural and genetic integrity [65, 66]. An abrupt stress response is often followed by a long term physiological stabilization over multiple generations representing selection for cellular processes under the new condition [66]. In *E*. *huxleyi* populations, this is likely the case [31] although an *E*. *huxleyi* strain has been reported to display short-term responses in 26 hours (~1 generation) that are comparable to responses in cells acclimated in batch and continuous cultures over many days and generations [67]. These acclimating strategies give organisms a physiological advantage over other organisms that have not been exposed to that environment [68], although biota can respond to chronic stress by reducing fitness [69]. In a modeling study, [70] explored the biogeochemical implications of this intraspecific variability by studying trends in the PIC:POC ratio in *E*. *huxleyi*. They suggested that *E*. *huxleyi* displayed comparably broad physiological properties and that the different nutrient and light regimes rather than strain-specific responses determine the outcome of this biogeochemically important proxy for carbon uptake. Our data do not strongly support this hypothesis but rather indicate that the intraspecific physiological properties may drive the selection of strains under varying conditions. This has been postulated by [52], who argued that *E*. *huxleyi* is universally distributed but that local selection of strains in largely monospecific populations explains their adaptability to changing environmental selection pressure. Field studies also support strain selection as a possible explanation for biogeographic or temporal patterns of strains associated with distinct CO_2_ conditions (e.g., [60, 62].

### Short-term physiological shifts in *E*. *huxleyi* NZEH

In our incubations, we achieved a distinguishable gradient in concentrations of CO_2_, HCO_3_^-^, CO_3_^2-^ and pH, with consistent increases in [CO_2_] and [HCO_3_^-^] and decreases in [CO_3_^2-^] and pH with depth (Table 2). Temperature was kept constant in all incubations and a difference of <10% in salinity between the different depths (Table 1) is unlikely to have caused an effect on cellular growth [71]. It is possible that shifts in physiological performance may be due, at least in part, to varying concentrations of inorganic carbon species. The observed lower growth rate and an increase in the cellular standing stocks of carbon (PIC and POC) and PON, with increasing [CO_2_] (12%) and decreasing pH (0.03–0.04 units) in the deep water incubations (Tables 2 and 3) are in agreement with previous laboratory results using strain NZEH, although our data show a much greater magnitude of response. Specifically, our study identified ~50% lower growth rates with 12% increase in [CO_2_] compared to smaller differences in growth rate observed by [55] (30%), [45] (20%) and [31] (19%) with greater differences and values of pCO_2_ between treatments. Some studies have reported no effect of CO_2_ on the growth in this strain [57, 61], although all these experiments used different CO_2_ levels, and there is substantial uncertainty of measured and calculated carbon chemistry parameters, particularly under high CO_2_ levels [72]. Although the cellular stocks of PIC, POC and PON increased with increasing CO_2_ and nutrient availability, the production rates of PIC were lower after incubation in the deep water (Table 3), in contrast with previous studies [55, 57]. These discrepancies can be explained by the far greater decline in growth rates observed in this study when cells were incubated in the deepest water, analogous to results obtained for this strain by [31].

For strain CCMP 88E, production rates of POC and PON were comparable in seawater from 1,000 and 4,800 m, the POC:PON ratio was lower at 4,800 m and the PIC:POC ratio remained unaltered (Table 3). Some of these results (POC:PON) differ from previous reports on *E*. *huxleyi* NZEH [31, 55, 57, 73]. We also note that this may be a consequence of strain variability, since strain NZEH exhibited no difference in PIC:POC or POC:PON ratios, consistent with previous observations [31, 55]. Interestingly, a modeling study by [70] proposed that responses in the PIC:POC ratio in *E*. *huxleyi* are highly plastic under varying carbonate conditions and that these responses can be predicted by the seawater concentrations of CO_2_, total alkalinity, and phosphate conditions, although this has not been validated in the field. The PIC:POC ratio is a particularly important proxy because it reveals the direction of CO_2_ fluxes between the atmosphere, the upper ocean, and the ocean interior in the short term [74] and on geological time scales [75]. Our results indicate that, although cellular quotas of PIC and POC increase in strain NZEH with increasing CO_2_, the lack of variance in PIC:POC for either NZEH or 88E means it is unlikely that carbonate chemistry alterations induced by artificial upwelling will result in significant changes in coccolithophore CO_2_ sequestration.

### Ecological and biogeochemical implications

Our approach explores the net physiological shifts after 72 hours of exposing *E*. *huxleyi* strains to seawater from varying depths, an approach that has been suggested to have the potential to fertilize the surface ocean [76, 77], although the value of this approach to drawdown CO_2_ has been questioned [78]. Specifically, [76] proposed the use of ocean pipes with the aim of enhancing photosynthetic carbon fixation by fertilizing the surface ocean with deep waters containing elevated nutrients and CO_2_ concentrations. A major criticism of this scheme is that it relied on the removal of CO_2_ by primary production, which is likely counteracted by degassing of CO_2_ as deep waters reach the sea surface. In addition to this, there is the issue of decoupling between productivity at the sea surface and carbon export at depth; although carbon fixation will likely occur, the mechanism by which carbon is subsequently exported to depth remains an open question.

Very few studies have been able to confirm whether enhanced photosynthetic carbon fixation and biomineralization translates into an increase in carbon export below 1,000 m on centennial time scales (carbon sequestration as defined by IPCC [79]) [19, 80]. Additionally, the response of ecosystems and the structure and productivity of the microbial community is likely to vary with geographic location, season, upwelled water composition, and the rate and phasing of upwelling [80]. In our experiment, there was no difference in POC production for either strain when incubated in waters from 1,000 and 4,800 m. However, for strain NZEH, we observed a decline in growth rates when using the deepest waters (which contained more CO_2_) and a concomitant increase in cellular calcification, These data are in agreement with previous observations for this strain when incubated under high CO_2_ conditions [31, 55]. Increases in cellular calcification may result in energetic trade-offs in calcifying organisms [81, 82, 83] and could lead to competitive disadvantages [31]. These physiological changes alongside changes in POC:PON (which decreased significantly with increasing CO_2_ levels in CCMP 88E but remained unaltered in NZEH) have the potential to alter grazing selection [84]. Cell volume appeared to remain unaltered in CCMP 88E but increased by 30% with increasing CO_2_ (in 4,800 m water) in NZEH (Fig 1), in agreement with other studies showing similar increases in coccosphere volume ([55]: 21%; [31]: 30%). A decrease in ecological fitness as evidenced by declining growth rates in the overcalcified strain (NZEH) and changes in cell volume could affect grazing pressure by zooplankton [85, 86]. This indicates that the suggested geoengineering approaches may have unexpected and unpredictable outcomes because of the genomic and physiological variability within the phytoplankton species concept.

In addition to macronutrients, deep seawater rising to the euphotic zone can carry micronutrients such as Fe [87, 88] and Zn [89]. These metals are involved in carbon fixing mechanisms by controlling the activity of metalloenzymes such as carbonic anhydrase [90]. Our study area in the North Atlantic Ocean is a region of low deep water Fe concentrations [91] and it seems unlikely that micronutrient limitation had a significant influence on our experiments. Increases in micronutrients are associated with increasing productivity [92], which in turn controls the flux of organic carbon export and thus the CO_2_ drawdown in the surface ocean on ecological [93] and geological time scales [94, 95]. For example, Fe availability may be important in regulating coccolithophore bloom formation in cold, nutrient-rich waters of the Patagonian Shelf, where iron may be supplied by shelf sediments [96, 97]. However, the distribution of elements and their ratios is complex and known to vary depending on physical conditions. For example, horizontal and vertical changes in the seawater Sr/Ca ratios can vary by up to 3% [98], which translates into a change in the calcite Sr/Ca ratios in the calcite produced and exported to depth.

A few coastal studies have used natural CO_2_ gradients in the field as analogues of ocean acidification; e.g., the CO_2_ flux from volcanic vents that lowers coastal seawater pH in the Tyrrhenian Sea, from current levels (~8.1) to levels beyond worse case scenarios (~7.4) [99]. These studies suggest that variations in CO_2_ can cause species-specific as well as whole community responses [100]. Results from most of these experiments indicate that calcification is detrimentally affected by elevated CO_2_; e.g., in epiphytic coralline algae cover and mass calcification on sea grass meadows [101]. However, in other groups (bryozoans), calcification can be maintained under decreased pH levels but an additional stressor such as warming, can interrupt calcification [102]. Therefore, on top of inter- and intraspecific inherent properties, multi stressor and synergistic impacts are likely to be responsible for the observed different responses of coccolithophores to water from varying depths.

### The elemental composition of seawater and geochemistry of coccoliths

Several factors are known to influence the elemental ratios used in this study. These include temperature, growth rates and calcification rates. For example, in coccolithophores, calcite Sr/Ca ratios increase by 1–2% per 1°C increase in temperature [103] as well as in foraminifera [104]. In the current study, the calcite Sr/Ca was depth-dependent despite constant seawater Sr/Ca, and showed intraspecific variability. This could be caused by a decrease in the discrimination of Sr relative to Ca, during the calcification process [105]. The observed change in coccolith calcite Sr/Ca with depth, and therefore increasing DIC, occurs in laboratory CO_2_ manipulations for both coccolith calcite Sr/Ca and Mg/Ca ratios [106]. We can speculate than in other upwelling regions where deep waters have also a different seawater Sr/Ca and DIC than surface water, the calcite Sr/Ca could also change. In practical terms, this means that the reconstruction of paleo-oceanographic variables using coccolith calcite Sr/Ca is complicated by the influence of different carbonate chemistries of the original water mass on the coccolith calcite Sr/Ca. Both rising temperature and CO_2_ are known to increase coccolith calcite Sr/Ca [106], and if we add this to observed difference in seawater Sr/Ca of up to 3% on horizontal transects (Atlantic and Pacific Oceans) [98], the use of this ratio as a paleo tool to reconstruct productivity can be compromised.

## Concluding remarks

Although any extrapolation to the field would require *in-situ* testing of the effect of upwelling on coccolithophore performance and population dynamics as a baseline for comparison, our observations revealed some interesting trends. For example, our data show that use of the deepest waters (~4,800m) enhanced cellular PIC and POC in one strain of *Emiliania huxleyi*, suggesting that deep upwelling might be useful to promote carbon fixation by coccolithophores at the ocean surface. Although we only investigated two strains, growth was not promoted for either strain using water from <1000m, implying that shallow water geoengineering may not be suitable for all locations, and any such approach should be investigated on a case-by-case basis. The strain- and depth-specific variability revealed in this study highlights how, without careful foresight, there are likely to be substantial challenges when designing and applying universal geoengineering approaches to enhance carbon sequestration and promote other microbial biogeochemical responses.

## Supporting information

S1 Table Irradiance during the incubation periods. Photosynthetically active radiation in μmol quanta m^-2^ s^-1^ over the 24 hour cycle during the incubation period.
(DOCX)

S2 Table Inorganic carbon chemistry parameters. Initial (t_0_) and final (t_72_) conditions are provided. First row indicates the values of the water at t_0_, immediately after filtration; the second row indicates the values at the end of the experiment (t_72_); the third row represents one standard deviation; and the last row represents the values in the blank (seawater treated in an identical way but without cells).
(DOCX)

S3 Table Carbon chemistry parameters before (white) and after (gray) filtration. TA = total alkalinity; DIC = dissolved inorganic carbon. TA, DIC, HCO_3_^-^, CO_3_^2-^ and CO_2_ values are in μmol kg seawater^-1^. pCO_2_ = partial pressure of CO_2_ values are in parts per million volume.
(DOCX)
