# Supplementary material for: Physiological responses of coccolithophores to abrupt exposure of naturally low pH deep seawater
Source: PLoS One. 2017 Jul 27;12(7):e0181713. doi: 10.1371/journal.pone.0181713 (PMC5531516; doi:10.1371/journal.pone.0181713)
Supplement: S1 Table — Photosynthetically active radiation in μmol quanta m-2 s-1 over the 24 hour cycle during the incubation period. (DOCX) [file pone.0181713.s001.docx]

| **PAR (μmol quanta m^-2^ s^-1^)** | **%** |
| --- | --- |
| <150 | 20.2 |
| 150-300 | 14.7 |
| 300-1000 | 46.2 |
| 1000-2000 | 18.3 |
| >2000 | 0.6 |
