# Supplementary material for: Physiological responses of coccolithophores to abrupt exposure of naturally low pH deep seawater
Source: PLoS One. 2017 Jul 27;12(7):e0181713. doi: 10.1371/journal.pone.0181713 (PMC5531516; doi:10.1371/journal.pone.0181713)
Supplement: S2 Table — Initial (t0) and final (t72) conditions are provided. First row indicates the values of the water at t0, immediately after filtration; the second row indicates the values at the end of the experiment (t72); the third row represents one standard deviation; and the last row represents the values in the blank (seawater treated in an identical way but without cells). (DOCX) [file pone.0181713.s002.docx]

| **CCMP 88E (I1)** | **TA (µmol kg SW^-1^)** | | **DIC (µmol kg SW^-1^)** | **pH** | | **[HCO_3_^-^] (µmol kg SW^-1^)** | **[CO_3_^2-^] (µmol kg SW^-1^)** | **[CO_2_] (µmol kg SW^-1^)** | **Ω-cal** | **pCO_2_ (p.p.m.v)** |
| --- | --- | --- | --- | --- | --- | --- | --- | --- | --- | --- |
| 4757 m (t_0_)  (t_72_)  Blank | 2340.87  2340.79  (2.70)  2348.70 | | 2203.39  2169.24  (17.73)  2187.80 | 7.90  7.92  (0.04)  7.898 | | 2070.62  2018.48  (25.69)  2041.90 | 108.37  130.12  (10.07)  123.80 | 24.40  20.64  (2.04)  22.10 | 2.58  3.10  (0.24)  2.95 | 595.77  574.38  (56.71)  614.40 |
| 1002 m (t_0_)  (t_72_)  Blank | 2343.43  2369.96  (2.08)  2375.10 | | 2181.75  2187.23  (1.71)  2188.90 | 7.93  7.94  (0.00)  7.943 | | 2037.11  2029.99  (1.51)  2029.80 | 123.14  137.27  (0.43)  139.50 | 21.50  19.97  (0.06)  19.60 | 2.92  3.25  (0.01)  3.31 | 557.05  558.72  (1.63)  549.60 |
| 502 m (t_0_)  (t_72_)  Blank | 2346.25  2346.39  (0.26)  2347.40 | | 2135.83  2131.12  (7.54)  2135.30 | 8.00  8.01  (0.01)  8.01 | | 1964.95  1958.03  (11.42)  1963.90 | 154.20  156.87  (4.53)  154.90 | 16.69  16.21  (0.65)  16.50 | 3.66  3.72  (0.11)  3.68 | 471.87  453.04  (18.08)  461.30 |
| 10 m (t_0_)  (t_72_)  Blank | 2347.37  2360.59  (0.40)  2362.40 | | 2113.76  2093.42  (1.80)  2092.40 | 8.07  8.11  (0.00)  8.11 | | 1931.36  1890.85  (3.07)  1888.10 | 167.92  190.07  (1.41)  192.00 | 14.49  12.50  (0.13)  12.30 | 3.98  4.51  (0.03)  4.55 | 391.27  349.65  (3.74)  345.20 |
| **NZEH**  **(I2)** | | **TA (µmol kg SW^-1^)** | **DIC (µmol kg SW^-1^)** | | **pH** | **[HCO_3_^-^] (µmol kg SW^-1^)** | **[CO_3_^2-^] (µmol kg SW^-1^)** | **[CO_2_] (µmol kg SW^-1^)** | **Ω-cal** | **pCO_2_ (p.p.m.v)** |
| 4831 m (t_0_)  (t_72_)  Blank | | 2334.05  2323.25  (1.46)  2348.18 | 2193.30  2160.62  (5.21)  2191.45 | | 7.91  7.90  (0.01)  7.88 | 2059.53  2014.54  (7.56)  2047.27 | 109.97  124.53  (2.97)  121.44 | 23.80  21.55  (0.53)  22.74 | 2.62  2.97  (0.07)  2.90 | 581.02  609.89  (7.52)  639.23 |
| 1010 m (t_0_)  (t_72_)  Blank | | 2356.85  2293.18  (4.87)  2342.73 | 2187.46  2129.32 (5.07)  2178.01 | | 7.95  7.91  (0.01)  7.89 | 2038.69  1984.11  (4.91)  2029.88 | 127.97  124.27  (0.13)  126.31 | 20.81  20.94  (0.24)  21.81 | 3.04  2.95  (0.00)  3.00 | 539.71  584.39  (16.76)  623.36 |
| 508 m (t_0_)  (t_72_)  Blank | | 2346.42  2332.18  n.d.  2341.82 | 2140.53  2153.16  n.d.  2165.71 | | 7.99  7.94  n.d.  7.94 | 1972.06  1999.18  n.d.  2012.93 | 151.34  134.28  n.d.  132.73 | 17.14  19.70  n.d.  20.05 | 3.59  3.19  n.d.  3.15 | 484.71  545.94  n.d.  550.55 |
| 38 m (t_0_)  (t_72_)  Blank | | 2352.50  2324.08  (13.17)  2340.48 | 2108.82  2109.19  (17.99)  2137.67 | | 8.08  8.01  (0.06)  7.99 | 1920.56  1936.90  (34.12)  1971.19 | 174.43  156.20  (18.79)  149.19 | 13.83  16.09  (2.39)  17.29 | 4.14  3.71  (0.45)  3.54 | 373.71  448.40  (69.79)  484.09 |
