# Supplementary material for: Physiological responses of coccolithophores to abrupt exposure of naturally low pH deep seawater
Source: PLoS One. 2017 Jul 27;12(7):e0181713. doi: 10.1371/journal.pone.0181713 (PMC5531516; doi:10.1371/journal.pone.0181713)
Supplement: S3 Table — TA = total alkalinity; DIC = dissolved inorganic carbon. TA, DIC, HCO3-, CO32- and CO2 values are in μmol kg seawater-1. pCO2 = partial pressure of CO2 values are in parts per million volume. (DOCX) [file pone.0181713.s003.docx]

|  | **TA** | **DIC** | **pH** | **HCO_3_^-^** | **CO_3_^2-^** | **CO_2_** | **Ω_Ca_** | **pCO_2_ (p.p.m.)** |
| --- | --- | --- | --- | --- | --- | --- | --- | --- |
| 4,800 m, I1 | 2354.3 | 2200.9 | 7.933 | 2061.0 | 117.7 | 22.3 | 2.81 | 543.3 |
| 4,800 m, I1 | 2327.2 | 2183.2 | 7.913 | 2048.4 | 111.6 | 23.2 | 2.66 | 566.3 |
| 4,800 m, I2 | 2363.4 | 2205.3 | 7.944 | 2062.7 | 120.9 | 21.7 | 2.88 | 529.9 |
| 4,800 m, I2 | 2340.9 | 2203.4 | 7.895 | 2070.6 | 108.4 | 24.4 | 2.58 | 595.8 |
| 1,000 m, I1 | 2369.9 | 2193.6 | 7.949 | 2041.1 | 132.3 | 20.2 | 3.13 | 524.7 |
| 1,000 m, I1 | 2370.3 | 2193.2 | 7.951 | 2040.3 | 132.8 | 20.1 | 3.15 | 522.4 |
| 1,000 m, I2 | 2345.5 | 2182.3 | 7.926 | 2036.8 | 124.1 | 21.3 | 2.95 | 552.5 |
| 1,000 m, I2 | 2343.4 | 2181.8 | 7.923 | 2037.1 | 123.1 | 21.5 | 2.92 | 557.0 |
| 500 m, I1 | 2345.9 | 2142.8 | 7.972 | 1975.9 | 149.5 | 17.4 | 3.55 | 492.6 |
| 500 m, I1 | 2346.6 | 2145.2 | 7.968 | 1979.2 | 148.5 | 17.6 | 3.53 | 497.6 |
| 500 m, I2 | 2346.2 | 2134.4 | 7.991 | 1962.8 | 155.1 | 16.6 | 3.68 | 468.2 |
| 500 m, I2 | 2346.2 | 2135.8 | 7.988 | 1965.0 | 154.2 | 16.7 | 3.66 | 471.9 |
| ChlMax, I1 | 2361.4 | 2097.2 | 8.111 | 1897.0 | 187.7 | 12.5 | 4.45 | 338.8 |
| ChlMax, I1 | 2357.6 | 2103.9 | 8.093 | 1909.8 | 180.9 | 13.2 | 4.29 | 356.1 |
| ChlMax, I2 | 2349.5 | 2113.2 | 8.062 | 1929.2 | 169.7 | 14.3 | 4.03 | 386.3 |
| ChlMax, I2 | 2347.4 | 2113.8 | 8.057 | 1931.4 | 167.9 | 14.5 | 3.98 | 391.3 |
